# Practical Use of Starlink Downlink Tones for Positioning

**DOI:** 10.3390/s23063234

**Published:** 2023-03-18

**Authors:** Nabil Jardak, Ronan Adam

**Affiliations:** French-German Research Institute of Saint-Louis, 5 Rue du Général Casssagnou, 68300 Saint-Louis, France; ronan.adam@isl.eu

**Keywords:** positioning, signal of opportunity, Doppler shift, low earth orbit, Ku-band, Starlink

## Abstract

The large availability of Low Earth Orbit (LEO) satellite systems makes them useful beyond their original purposes, such as in positioning, where their signals can be passively used. In order to determine their potential for this purpose, newly deployed systems need to be investigated. This is the case with the Starlink system, which has a large constellation and is advantageous for positioning. It transmits signals in the 10.7–12.7 GHz band, the same as that of geostationary satellite television. Signals in this band are typically received using a low-noise block down-converter (LNB) and a parabolic antenna reflector. Regarding opportunistic use of these signals in small vehicle navigation, the dimensions of the parabolic reflector and its directional gain are not practical for tracking many satellites simultaneously. In this paper, we investigate the feasibility of tracking Starlink downlink tones for opportunistic positioning in a practical situation, when signals are received without a parabolic reflector. For this purpose, an inexpensive universal LNB is selected, and then signal tracking is performed to determine the signal and frequency measurement quality, as well as the number of satellites that can be tracked simultaneously. Next, the tone measurements are aggregated to handle tracking interruptions and to recover the traditional Doppler shift model. After that, the use of measurements in multi-epoch positioning is defined, and its performance discussed as a function of the relevant measurement rate and the required multi-epoch interval duration. The results showed promising positioning which can be improved by selecting a better-quality LNB.

## 1. Introduction

The localization of airborne systems such as drones is usually based on Global Navigation Satellite Systems (GNSS), which are vulnerable to interference as evident by the resurgence of incidents reported near commercial ports, on highways, and near civil aviation [1]. Hence, it is necessary to look for alternatives or augmentations that are capable of maintaining navigation when the GNSS service is degraded.

A large number of LEO constellations orbit around the Earth, typically for communications, broadband connectivity, scientific missions such as Earth and atmospheric observation, etc. LEO systems are just beginning to be introduced into smartphones for off-grid communications, such as Iridium, which is being integrated into a new chip from Qualcomm to enable satellite-based messaging and emergency calls in locations not covered by mobile networks [2]. This wide availability of LEO systems is also valuable for location-based applications due to the frequency diversity, the high signal strength, and the wide coverage area it provides. Several studies have investigated the use of LEO satellites for positioning, navigation, and timing (PNT) purposes [3], either as a dedicated system for navigation (i.e., LEO-PNT), or passively, where they are used as signals of opportunity (i.e., LEO-SOP). First, LEO-PNT has benefited primarily from academy research [4,5,6]. At present, a LEO-PNT system is being developed by Xona [7], while Iridium has been “augmented” with a special navigation message by Satelles [8] to enable location and time synchronization. In [9], a global navigation concept that uses carrier Doppler shift measurements of a large LEO constellation is investigated and shown to be a potential alternative to GNSS. LEO-SOP is the most prevalent in the literature thanks to the availability of operational systems such as Iridium-Next, Orbcomm [10], and Globalstar [11].

Early research projects using LEO systems for navigation have shown promising positioning results. In [12], experiments based on data from two Orbcomm satellites and assuming a known altitude exhibited a 2D positioning error of 358 m for a static user. In [13], an experiment using 4 min of data for 2 Orbcomm satellites have led to a positioning accuracy of a few hundred meters for a land vehicle. In [14], experiments showed a positioning accuracy of 25 m with Iridium in differential positioning mode in a static location.

Specifically, a review of the existing literature on LEO-SOP shows advanced work covering various positioning types. A first class of LEO satellite-based navigation techniques uses only LEO satellite signals in which the positioning can be either autonomous [15,16] or in differential mode [14,17]. It can be based on the carrier phase [18] or on Doppler-shift measurements [15,16]. A second class performs a coupling between LEO satellite measurements and an IMU (gyroscopes and accelerometers) [19,20] with magnetometers [21]. In general, tight coupling is considered due to the weak satellite visibility from operational LEO systems that makes it difficult to determine a standalone positioning. In both categories, navigation is either single- or multi-constellation, where measurements from two or more LEO systems are coupled to cope with the limited availability of LEO satellites from existing systems [12,22]. In general, operational constellations such as Iridium, Orbcomm, and Globalstar are used, however the advent of “super-constellations” such as Starlink is likely to improve positioning quality and availability. Recently, the use of Starlink as an SOP has gained interest thanks to rapid constellation deployment [23,24,25].

The Starlink downlink signal has several channels with large bandwidths (240 MHz) in the 10.7–12.7 GHz frequency band [26]. Within this band, there are multiple tones in a few bandwidths of ~1 MHz, which have been observed in many satellite signals. One of the barriers to using a new system as an SOP for navigation is the unknown structure of the signal, as it is usually not made public. In [25], the authors have identified the structure of the Starlink downlink signal, which should enable advanced measurements such as pseudo-distance on broadband Starlink signals.

In this paper, since we are targeting small platform localization with low power consumption, we will use Starlink downlink tones when received without an antenna parabolic reflector. The tones allow the carrier frequency to be tracked easily, which is useful for Doppler shift-based positioning. The relatively narrow bandwidth occupied by the tones (~1 MHz) can be handled by low-cost software defined radios (SDRs) and CPUs. Moreover, the signals in the 10–12 GHz band are in general captured with a parabolic reflector and a low-noise block down-converter (LNB). The parabolic reflector concentrates the downlink beam coming from a very narrow direction toward the LNB, and hence increases the signal power. However, it is cumbersome in terms of space for vehicles such as drones, and has a selective radiation pattern that prevents the acquisition of multiple satellites simultaneously. For these considerations, we will study tones tracking without a parabolic reflector. In this case, the signal will be much weaker than if it had been captured with a parabolic reflector, but the tone frequency measurements do not need as much power as those required, for instance, to decode useful data.

This paper aims to contribute to the current literature of LEO-SOP by taking a step forward in the potential use of Starlink satellite signals for opportunistic navigation. Although Starlink signal processing has been the focus of a few recent papers, many questions remain unanswered regarding the quality of the signal received on the ground, the quality of the measurements, and the number of satellites that can be tracked simultaneously. In this paper, we intend to address these questions, especially when using a universal down-converter without a reflector antenna.

Therefore, in this paper, we present the processing and characterization of Starlink downlink tones and the feasibility of their use without a parabolic reflector for positioning purposes. After the introductory section (Section 1), Section 2 describes the detection and tracking of real-life collected tones. Section 3 performs the aggregation of multi-tone measurements leading to a useful frequency shift model. Section 4 presents a practical use of tone frequency shifts in multi-epoch positioning. Section 5 is the conclusion.

## 2. Signal Detection and Tracking

### 2.1. Range Dynamics and Visibility

The current Starlink constellation consists of nearly 3000 satellites (in January 2023) placed in several low Earth orbit planes. The orbits are nearly circular and have three main inclinations: ~53.2°, 70°, and 97.7°. The first inclination is the most deployed today, which makes the satellites visibility better in equator and mid-latitude regions than near polar regions.

In order to determine the signal dynamics which are needed to design the signal processing, we simulated the Starlink constellation based on orbit parameters that are published daily by NORAD in two-line element (TLE) files [27]. We used the Simplified General Perturbation Model, SGP-4 [28,29], to compute the positions and velocities of the satellites. This allowed us, for a given location of the observer, to derive typical values for the dynamics of the distance between the satellite and the user, as well as the expected visibility of the satellites (Table 1).

The Doppler shift and Doppler shift rate have high values resulting from the high speed of LEO satellites combined with the use of signals in the Ku band. The high visibility of the satellites is a geometric visibility (i.e., the number of satellites in view simultaneously over an elevation mask). Effective visibility, which is defined relative to the minimum received signal strength that allows tracking of the signals, will be much lower due to the low altitude of the satellite orbits. In addition, the low orbit altitude increases the satellite speed and reduces the time the satellite remains above a given elevation mask for near-ground observers. A satellite remains in view above an elevation angle of 25° for only a few minutes.

### 2.2. Signal Recording

The user’s downlink signal is in the 10.7–12.7 GHz band, which is the same band used by geostationary television satellites. The signal is broken down into several sub-bands spaced 250 MHz apart [25], within which there are multiple tones in a bandwidth of nearly 1 MHz. In this work, we will use the tone band centered on Fo = 11,325 MHz.

The reception of Ku-band signals can classically be done with a reflector-based or a parabolic dish antenna. These types of antennas offer the best performance in terms of bandwidth and gain, but they require motorization in order to track the satellites over the whole field of view, making it difficult if not impossible to mount them on a small vehicle or flying platform such as a UAV. An example of such an antenna is General Dynamics’s M20-24M Ku Satcom On-The-Move antenna, whose volume and weight are approximately 0.3 cubic meters and 59 kg, respectively. Moreover, due to the typical beamwidth of these antennas, it is only possible to track a single satellite at a time.

On the other hand, electronically scanned phased array antennas offer the smallest profile, at the expense of design and computational complexity, power consumption, and cost, which limits their application in most consumer products. They are also theoretically capable of tracking multiple satellites. Examples of commercially available user’s phased arrays for the tracking of LEO signals are Hanwha Phasor’s antenna, Requtech’s REqRESA-S Ku antenna, and Starlink user’s dish antenna [30].

The selection of the antenna and of the LNB has been carried out considering constraints of size, weight, and cost. A key feature is the antenna beamwidth, which should be sufficiently large to simultaneously detect most of the satellites in view. This choice is made at the expense of the antenna gain and therefore of the signal-to-noise ratio in order to capture many satellites and optimize the satellites’ geometry. Universal LNBs have reduced size and weight, and can be easily obtained at a lower cost. They also offer a reasonable compromise between beamwidth and gain.

**Figure 1:**
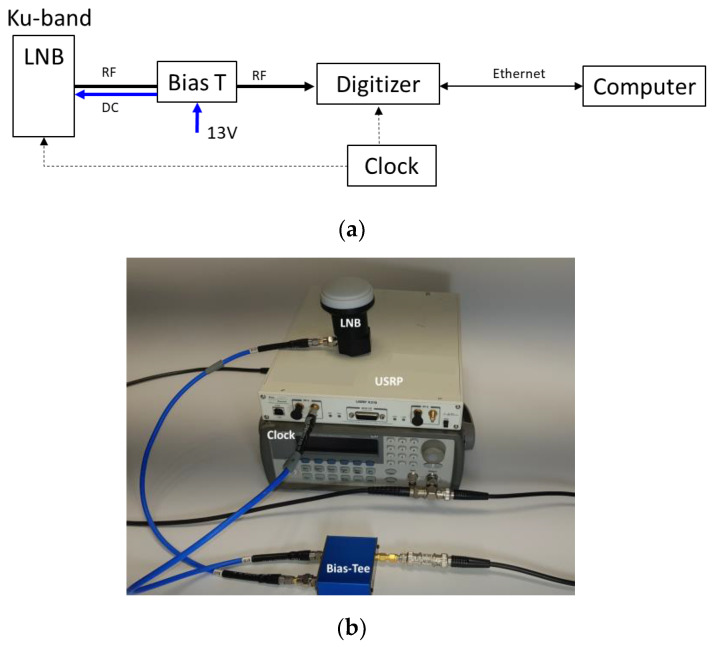
(**a**) Sketch of the recording chain, LNB down-convertor used without a parabolic reflector. (**b**) Photo of the experimental set-up.

We have set up an acquisition tool to record the Starlink downlink tones around Fo (Figure 1a). A universal Ku-Band satellite LNB usually dedicated to satellite TV reception is employed to receive and down-convert the Ku band signals to a lower frequency (typically around 1–2 GHz). It has a local oscillator frequency of 9750 MHz. As it is supposed to be mounted on a parabolic dish antenna, it also directly integrates a linearly polarized choked-feed horn antenna with a half-power beam width of around 35°. The LNB provides a 50 dB gain conversion. However, since the Starlink downlink signal would have a circular polarization (RHCP), a mismatched loss of 3 dB is expected to occur when using this LNB. A bias-tee and a DC power supply is used to power the LNB. It is then connected to the USRP X310. An external 10 MHz reference is provided by Keysight’s 33,250. A waveform generator (whose TXCO have a frequency accuracy of 2 ppm) is employed to discipline both the digitizer and the LNB clocks. A photo of the experimental set-up is provided in Figure 1b. The recorder has a signal timestamp capability of a few seconds. Finally, we emphasize the absence of a parabolic reflector that is traditionally associated with the LNB to capture Ku band signals.

**Figure 2:**
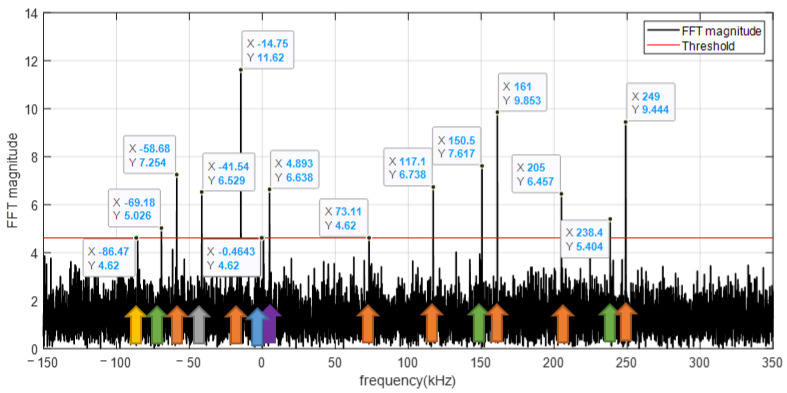
FFT magnitude centered at 11,325 MHz. The arrows of the same color indicate the tones transmitted by the same satellite. Four satellites are captured in total, with a number of tones varying from 1 to 6.

We recorded real-life data at a static location on the rooftop of a building to be near open-sky reception conditions. The LNB down-converts the 11,325 MHz carrier to 1575 MHz. The acquisition software was configured to sample the signal with a sampling rate of 2 MHz. Figure 2 gives an example of the spectrum of the recorded data computed over a signal burst of 14 ms. It shows that despite the absence of a parabolic reflector, many tones occupy the frequency interval surrounding 11,325 MHz with a magnitude well above the noise floor. The tones that are spaced ~44 kHz apart belong to the same satellite (indicated by arrows with the same color on the figure) and have different magnitudes. Typically, between one and several tones are captured for a satellite. We will show later that nine tones can be received for a single satellite.

### 2.3. Tones Detection and Tracking

In this section, we track the tones to measure their frequency shifts, which can then be used for positioning. We assume that the incoming signal is formed by perfect tones (i.e., un-modulated carriers).

**Figure 3:**
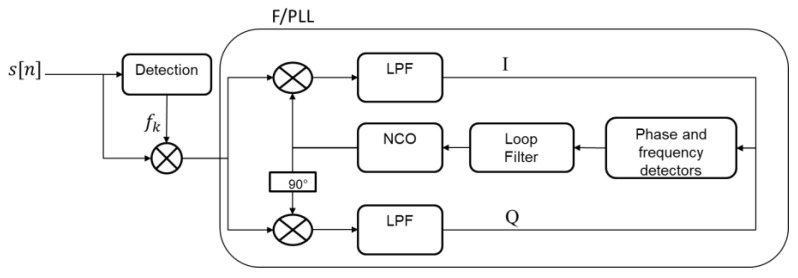
Principle of the tone detection and tracking.

Figure 3 presents the principle of detection and tracking of the phase and frequency of the tones. We perform the detection by identifying the spectrum magnitude components that exceed a given threshold, i.e.,$\left|X_{f}\left(f\right)\right|>Xo$, where $\left|X_{f}\left(f\right)\right|=\sqrt{Re\left(X_{f}\right)²+Im\left(X_{f}\right)²}$ is the magnitude of the normalized Fourier transform of the received signal and Xo is a threshold. With the assumption that only tones occupy the sampled frequency band, when they are absent, the signal distribution is Gaussian and so do its FFT real and imaginary components. As a result, the spectrum magnitude follows a Rayleigh distribution, and the threshold can be designed according to $Xo=F_{R}^{-1}\left(1-PFA,\sigma \right)$, where $F_{R}$ is the cumulative density function of the Rayleigh distribution with parameter $\sigma =E\left(\left|X_{f}\left(f\right)\right|\right)/\sqrt{\pi /2}$, with E(.) being the expectation operation, and PFA as a probability of false alarm.

**Figure 4:**
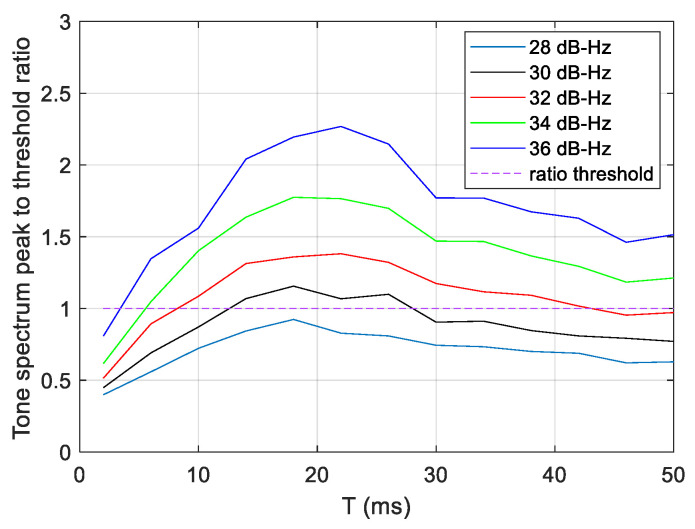
Ratio between the tone spectrum peak and the detection threshold as a function of the duration of the FFT burst, T, and for different C/No levels. A tone dynamic of 5 kHz/s is assumed.

The expected weak signal level suggests that computing the FFT over a long burst would reduce the noise, however the FFT length is limited by the high-frequency shift rate. This is because over a long time interval, the tone frequency scrolls through several FFT frequency bins, and thus the signal energy is spread over many bins. In addition, long FFTs consume a lot of resources in a practical implementation. In order to select an adequate size of FFT, we computed numerical simulations of the ratio between the tone spectrum peak and the detection threshold as a function of the duration of the FFT burst, T, and for different C/No levels (Figure 4). We can verify that at the beginning, the ratio increases with T, reaches a maximum, and then decreases. The tone spectrum peak is below the detection threshold (i.e., ratio < 1) for C/No = 28 dB-Hz whatever the duration of the burst is, while for C/No higher than 30 dB-Hz, the detection becomes possible for a range of T values. For 30 dB-Hz, suitable values of T are between ~14 and 26 ms. Therefore, 14 ms is an acceptable compromise for the FFT burst duration. It should be noted that the interval of T for which the detection is possible becomes wider when the C/No increases, and in particular, the detection is possible using small values of T (down to ~4 ms for C/No = 36 dB-Hz).

For each detected tone that is not being tracked, the signal is demodulated by the detected frequency and then supplied to the frequency and phase tracking module. The tracking process implements a third-order phase-locked loop (PLL) assisted by a second-order frequency-locked loop (FLL) [31]. For convenience, we refer to the PLL and FLL combination as FPLL, whose principle is as follows: An NCO (numerically controlled oscillator) generates a complex local carrier over duration T that is multiplied by a burst of the incoming signal of the same duration. The results on the real and imaginary arms are then filtered by a low-pass filter (LPF) (a summation over T, called integration time) to obtain a complex signal ($I_{n}+jQ_{n}$), whose phase is the difference between the phase of the local carrier and the incoming tone. The resulting signal is used by a phase discriminator $\phi_{e}={\left(2\pi \right)}^{-1}\mathrm{atan}2\left(Q_{n},I_{n}\right)$ and by a frequency discriminator $f_{e}={\left(2\pi T\right)}^{-1}\mathrm{atan}2\left(Q_{n}I_{n-1}-Q_{n-1}I_{n},I_{n}I_{n-1}+Q_{n}Q_{n-1}\right)$ to derive the phase error $\phi_{e}$ and the frequency error $f_{e}$, respectively, between the incoming carrier and the local carrier. In order to reduce noise and produce an accurate estimate of the phase and frequency of the input signal, the phase error is then filtered by a second order loop filter, $F_{pll}\left(s\right)=\left(\omega_{0p}^{3}+1.1\omega_{0p}^{2}s+2.4\omega_{0p}s^{2}\right)/s^{2}$, where $\omega_{0p}$ is the natural frequency of the PLL that is related to the PLL equivalent noise bandwidth $\omega_{0p}={\left(0.7845\right)}^{-1}\;B_{0p}$; and the frequency error is filtered by a first order loop filter $F_{fll}\left(s\right)=\left(\omega_{0f}^{2}+1.414\omega_{0f}s\right)/s$, where $\omega_{0f}$ is the natural frequency of the FLL that is related to the FLL equivalent noise bandwidth $\omega_{0f}={\left(0.53\right)}^{-1}\;B_{0f}$. In the implementation, both loops share the same integrators. The FLL frequency error is injected one integrator back from the PLL, since the frequency error is in units of hertz while the phase error is in unit of cycles. The filter output is then integrated into the NCO ($F_{nco}\left(s\right)=1/s$), which adjusts the phase and frequency of the local carrier so that they match the phase and the frequency of the incoming tone. The NCO output is then mapped to cosine and sine functions, which represent an accurate replica of the incoming signal carrier. In general, since it has a large pull-in range (i.e.,1/T), the FLL is used at the beginning of the tracking process when the frequency error is large; then, after achieving frequency lock, both loops are activated until phase lock is achieved. At this time, the FLL is deactivated and only the PLL is used to keep precise carrier phase tracking. However, for applications with high dynamic stress, the FLL must be maintained over the whole tracking time. The FPLL is traditionally used to track L-band GNSS signal carriers that have a Doppler shift rate of 1 Hz/s (order of magnitude for a static user). We will show that it works for tracking LEO satellite signals in the Ku-band, which have a Doppler shift rate of a few kHz/s.

Successful locking of the tracking loops is achieved by keeping the resulting tracking error of the PLL and FLL within the operational ranges of their detectors. The tracking error is caused by thermal noise, dynamic stress, and for PLLs, oscillator-related errors. The discussion below will focus on the first two errors. The error due to thermal noise is inversely proportional to both the integration time, T, and to the received signal carrier-to-noise-density ratio C/No. It is also directly proportional to the equivalent noise bandwidth $B_{0}$, while the dynamic stress depends only on $B_{0}$. Thus, $B_{0}$ and T have to be designed such that the tracking loop remains stable in the intended application environment (i.e., dynamic stress and C/No). Below, we provide the values of $B_{0}$ and T and explain the reasoning behind the chosen values.

First, a third-order-PLL and a second-order-FLL are sensitive to jerk (i.e., $1/\lambda \times d^{3}R/dt^{3}$ ($\mathrm{Hz}/s^{2})$, where R is the range between the satellite and the user antennas). For a jerk of 70 Hz/s^2^ (Table 1), the dynamic stress error of a third-order-PLL is $70/{\left(B_{0p}/0.7845\right)}^{3}$ [cycle], and that of a second-order-FLL is $70/{\left(B_{0f}/0.53\right)}^{2}\;[$Hz]. When we use signals of opportunity of LEO satellites, and appropriate values of $B_{0p}$ and $B_{0f}$, such tracking errors are small compared to the error induced by the inaccurate orbit and clock, as we will see later. Note that by increasing $B_{0}$, the dynamic stress error is reduced, however the phase and frequency thermal noise errors increase.

On the other hand, the integration time T must satisfy a compromise between noise reduction (i.e., large value of T) and the ability to capture high frequency errors (i.e., short value of T). Indeed, in order to avoid a false frequency lock at the beginning of the tracking process, a wide pull-in range of the FLL is required, which is achievable with a short integration time, T.

Given these considerations, we set the integration time of the FPLL to T = 2 ms over the first 1-s of the tracking, then, we extend it to T = 10 ms until the signal loss of lock. We have found that an equivalent noise bandwidth of $B_{0p}=B_{0f}$ = 10 Hz is an acceptable compromise between noise and dynamic stress errors for a typical value of the expected C/No (30 dB-Hz). In this condition, we note that the dynamic stress error for the PLL is ~0.034 cycles, and that of the FLL is ~0.197 Hz.

**Figure 5:**
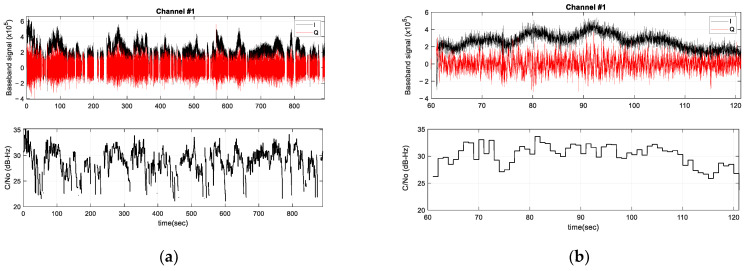
(**a**) Baseband signal (Phase arm (I) and Quadrature arm (Q)) and estimated C/No of the successively received satellite tones; (**b**) Zoom in showing a low signal level at the start and end times of satellite tracking, a high signal envelope at high satellite elevations, and a signal amplitude oscillation at low frequency (~0.1 Hz).

Many FPLL channels are instantiated to deal with receiving multiple tones. Tones from the same satellite are therefore tracked simultaneously on multiple channels. Figure 5 shows the resulting baseband signal and the estimated carrier-to-noise ratio (C/No) tracked by channel 1 for satellites that are received in succession during recording. Zooming in on the baseband signal (Figure 5b) shows that the signal envelope follows a bell shape, i.e., low amplitude toward low satellite elevation angles (i.e., when the satellite appears or disappears) and maximum power toward higher elevation angles where the satellite is closest to the observer. The oscillation in the signal amplitude has a period of ~10 s. This can be explained by the fact that the signal is received from different satellite sidelobes as the satellite moves.

The C/No of the tones is almost between 24 and 36 dB-Hz. This low signal level is due to the use of an LNB without a parabolic reflector. A parabolic reflector focuses the downlink beam to an open waveguide that feeds the LNB and thus adds a significant gain. It is interesting to evaluate tone tracking without the use of a parabolic reflector, because as mentioned above, the parabolic reflector is cumbersome for vehicles such as drones, and has a selective aperture that limits the number of satellites captured simultaneously. Phase array antennas can potentially track several satellites, but a large number of antenna elements are required to achieve parabolic reflector performances, so their cost and complexity are largely increased. Interestingly, measuring the tone frequency for localization does not require as much power as decoding useful data.

**Figure 6:**
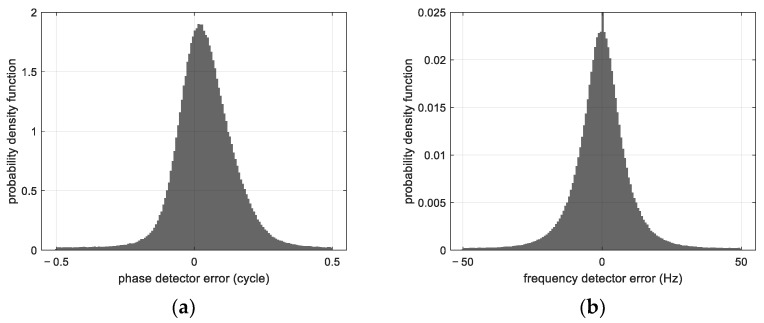
(**a**) Probability density function of the phase detector error, (**b**) Probability density function of the frequency detector error.

The probability density function of the phase detector error and that of the frequency detector error computed with the measurements of the tracked satellites are shown in Figure 6. The phase error has a standard deviation of 0.1157 cycles (3.1 mm), and is centered around 0.0384 cycles (1 mm). The standard deviation of the frequency detector error is 10.85 Hz (0.287 m/s) and is centered around −0.2337 Hz (−6 mm/s). For both detectors, the error distribution is not symmetrical, suggesting that the incoming signal does not satisfy the hypothesis of being formed by perfect tones. The non-zero average error can be explained by the dynamic stress error and by any distortion of the incoming signal.

### 2.4. Tones Frequency Shift

**Figure 7:**
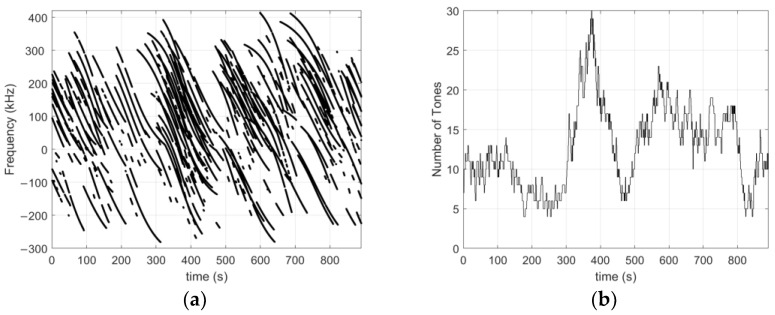
(**a**) Measured frequency of the tracked tones relative to the frequency Fo; (**b**) number of tracked tones.

Figure 7 shows the frequency offset from Fo measured on all channels. It shows that many tones are tracked simultaneously (between 4 and 30 tones at one time). Tone tracking lasts from a few seconds to two minutes, and is frequently interrupted. This is due to a weak signal strength that oscillates on both sides of the tracking sensitivity (oscillations are highlighted in Figure 5b).

**Figure 8:**
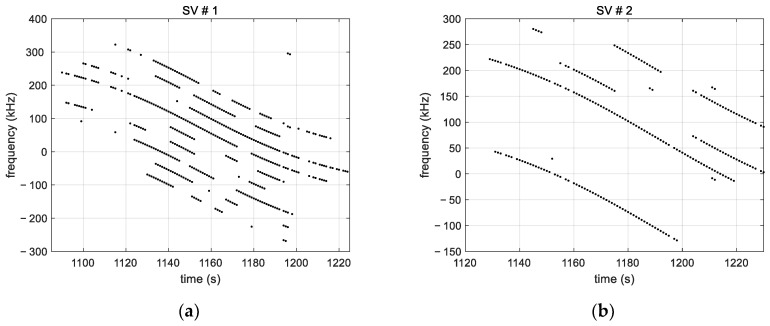
(**a**) Measured tone frequencies for satellite SV#1; (**b**) Measured tone frequencies for satellite SV#2, showing the difference in the number of tones tracked as well as the intermittent tracking. The latter is due to the low signal strength and the oscillating magnitude of the tones over time. The interruptions do not occur at the same time for tones of the same satellite because they have different amplitudes.

Figure 8 shows the measured frequencies for two satellites (SV). It can be seen that nine tones are captured in total for SV#1 and only five tones for SV#2. It further shows that tracking is frequently interrupted. This is a result of the weak signal strength of the tones combined with an oscillation in tone magnitude (observed in Figure 5). In other words, tones with a C/No that fluctuates around the tracking sensitivity (~23 dB-Hz) are alternately tracked and lost during the satellite visibility time, which explains the tracking interruptions. Furthermore, we note that the tracking interruptions do not necessarily occur at the same time for tones from the same satellite. This is due to the fact that tones from the same satellite do not have the same magnitude. It follows that high power tones are less likely to be frequently lost than low power tones. We further note that the tracking time of the tones is between a few tens of seconds and a few minutes.

**Figure 9:**
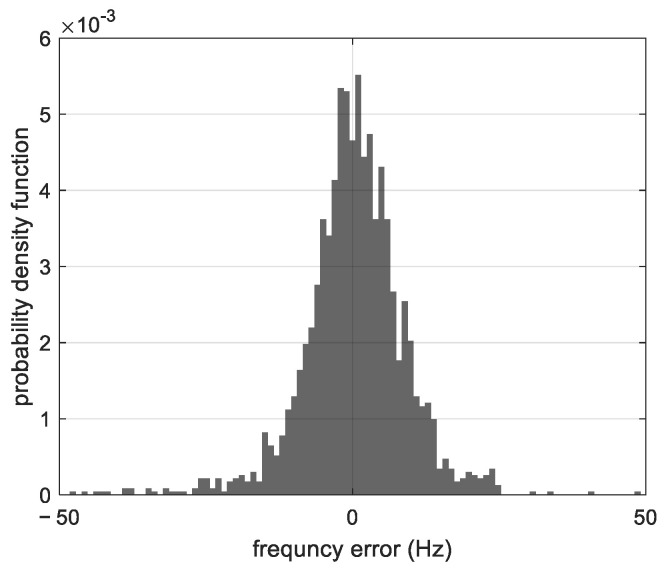
Probability density function of the frequency error of the tones.

Figure 9 gives the probability density function of the tones’ frequency error computed with the measurements of the tracked satellites. This error is the deviation of frequency measurement curves with respect to their polynomial fit. It shows a non-symmetrical distribution of the error with a standard deviation of 10.92 Hz (~0.28 m/s). The frequency tracking error is mainly caused by thermal noise (with an expected value of ~4.5 Hz (1σ) in the case of C/No = 31 dB-Hz, T = 10 ms, and $B_{0}$ = 10 Hz) and by the presence of distortions in the incoming signal that we observed during signal tracking.

## 3. Tones Frequencies Aggregation

In this section, we aggregate the tone measurements of the same satellite in order to cope with the frequent interruption of the measurement on each single tone, and to reduce the noise effect. For a tone transmitted at frequency Fo, the frequency shift measured at the user’s location is written

(1) $${\tilde{f^{k}}}_{Fo}\;≅f_{doppler}^{k}+f_{rx}-f_{sv}^{k}+f_{Tr}^{k}+\eta \prime^{k}$$

$f_{doppler}^{k}$ is the Doppler shift of the *k*th-satellite relative to Fo,

$f_{rx}$ is the receiver clock drift ($\dot{\delta t})\;$multiplied by Fo,

$f_{sv}^{k}$ is the *k*th-satellite clock drift (${\dot{\delta t}}^{k}$) multiplied by Fo,

$f_{Tr}^{k}$ is the troposphere induced frequency shift of the *k*th-satellite relative to Fo,

$\eta \prime^{k}$ is the measurement error due to thermal noise, interference, dynamic stress and signal propagation through the ionosphere. It is dominated by the thermal noise component if the interference effect is negligible.

For a tone transmitted at frequency Fo+N×ΔF, the frequency shift measured at the user’s location is written:

(2) $$\tilde{f^{k}}≅\left(f_{doppler}^{k}+f_{rx}-f_{sv}^{k}+f_{Tr}^{k}\right)\times \left(1+\frac{N\times \Delta F}{Fo}\right)+N\times \Delta F+\eta^{k}$$

ΔF=44 kHz is the frequency spacing between successive tones,

N is an integer which can take one of the nine values: −4, −3, −2, −1, 0, 1, 2, 3, 4,

$\eta^{k}$ is the measurement noise.

The goal is to estimate N in order to restore the frequency shift of each tone around Fo before aggregating the frequency shifts of all tones of the same satellite by taking their mean value at each epoch.

Since $N\times \Delta F/Fo<1.6\times 10^{-5}$, when we deal with the estimation of N, we can simplify the frequency measurement (2) to:

(3) $$\tilde{f^{k}\prime }≅f_{doppler}^{k}+f_{rx}-f_{sv}^{k}+f_{Tr}^{k}+N\times \Delta F+\eta^{k}$$

On the other hand, the predicted Doppler shift relative to measurement ${\tilde{f^{k}}}_{Fo}$ can be written:

(4) $${\hat{f}}_{doppler}^{k}≅f_{doppler}^{k}+f_{O}^{k}+f_{u}^{k}+f_{\Delta T}^{k}$$

where:

- $f_{doppler}^{k}$ is the *k*th-satellite Doppler shift (Hz)

(5) $$f_{doppler}^{k}=-\frac{1}{\lambda \;}\;\frac{{\left(r-r^{k}\right)}^{T}}{\left\|r-r^{k}\right\|}\left(v-v^{k}\right)$$

$\lambda =\frac{c}{Fo}$ is the carrier wavelength. r and v are the user’s position and velocity, respectively, in (m) and (m/s). $r^{k}$ and $v^{k}$ are *k*th-satellite position and velocity, respectively, in (m) and (m/s). ${\left(.\right)}^{T}$designates the transposition operator.

- $f_{O}^{k}\;$is the frequency shift induced by the orbit error for the *k*th-satellite (Hz). A rough approximation is

(6) $$f_{O}^{k}\approx \frac{1}{\lambda \;}\;\left(\frac{{\left(r-r^{k}\right)}^{T}}{\left\|r-r^{k}\right\|}\Delta v^{k}+\frac{{\left(v-v^{k}\right)}^{T}}{\left\|r-r^{k}\right\|}\Delta r^{k}\right)$$

where $\Delta r^{k}$ and $\Delta v^{k}$ are the *k*th-satellite position and velocity errors, respectively, in (m) and (m/s).

- $f_{u}^{k}$ is the frequency shift induced by the user position and velocity error for the *k*th-satellite. A rough approximation is

(7) $$f_{u}^{k}\approx -\frac{1}{\lambda \;}\;\left(\frac{{\left(r-r^{k}\right)}^{T}}{\left\|r-r^{k}\right\|}\Delta v+\frac{{\left(v-v^{k}\right)}^{T}}{\left\|r-r^{k}\right\|}\Delta r\right)$$

where Δr and Δv are the user’s position and velocity errors, respectively, in (m) and (m/s).

- $f_{\Delta T}^{k}\;$is the frequency shift induced by the timing error for the *k*th-satellite (Hz)

(8) $$f_{\Delta T}^{k}=\Delta T\times {\dot{f}}_{doppler}^{k}$$

where ΔT is the measurement timestamp error (user clock bias), and ${\dot{f}}_{doppler}^{k}$ is the Doppler shift rate (Hz/s).

Subtracting the measurement (3) from the prediction (4), then dividing the result by ΔF yields:

(9) $$\frac{\tilde{f^{k}}\prime -\hat{f^{k}}}{\Delta F}\approx \frac{f_{rx}-f_{sv}^{k}+f_{Tr}^{k}-f_{\Delta T}^{k}-f_{O}^{k}-f_{u}^{k}+\eta^{k}}{\Delta F}+N$$

If the contribution sum of all the errors is smaller than ΔF/2, i.e.,

(10) $$\left|f_{rx}-f_{sv}^{k}+f_{Tr}^{k}-f_{\Delta T}^{k}-f_{O}^{k}-f_{u}^{k}+\eta^{k}\right|<\frac{\Delta F}{2}\approx 22\;\mathrm{kHz}$$

then, for a given tone, the 44 kHz integer offset N can be calculated by taking the nearest integer value ⌊.⌋ of the difference between the measurement and its prediction divided by the tone spacing Δ*F*:

(11) $$N=\lfloor \frac{\tilde{f^{k}}\prime \;-\hat{f^{k}}}{\Delta F}\rfloor$$

In order to appreciate the validity of (10), we provide an example of the frequency error budget in Table 2.

It appears that the user clock drift, the user time error, and the satellite and user’s position errors are major contributors that affect the estimation of N. Note that a larger value of the user clock drift (for instance 2 ppm) leads to an error in the estimation of N, which is common to the tones, and as such is not actually an issue in Doppler-based positioning as it will be captured by the user clock drift state.

In case (10) is satisfied, the frequency shift of each tone of the *k*th-satellite, $\tilde{f^{k}}$, can be carried around Fo according to:

(12) $$\;\tilde{f_{o,N}^{k}}=\tilde{f^{k}}-\lfloor \frac{\tilde{f^{k}}\prime \;-\hat{f^{k}}}{\Delta F}\rfloor \Delta F$$

where $\tilde{f_{o,N}^{k}}\;$ is the measured tone frequency shift after the removal of the 44 kHz integer shift. The predicted Doppler frequency $\hat{f^{k}}$ of the *k*th-satellite is computed using (5) based on the user’s approximate location and velocity, the time of measurement, and the approximate satellite orbit on the day of collection (TLE files). Substituting (2) and (11) in (12) gives:

(13) $$\tilde{f_{o,N}^{k}}\;≅\left(f_{doppler}^{k}+f_{rx}-f_{sv}^{k}+f_{Tr}^{k}\right)\times \left(1+\frac{N\times \Delta F}{Fo}\right)+\eta^{k}$$

Then at each epoch, the frequency measurements of the tracked tones of the *k*th-satellite are converted around Fo before being averaged according to

(14) $$\tilde{f_{o}^{k}}\left(t\right)=\sum_{N}\tilde{f_{o,N}^{k}}\;\left(t\right)\times \left(\frac{Fo}{Fo+N\times \Delta F}\right)\;$$

This leads to the traditional single carrier frequency shift measurement model for the *k*th-satellite:

(15) $$\tilde{f_{o}^{k}}\;\left(t\right)≅f_{doppler}^{k}+f_{rx}-f_{sv}^{k}+f_{Tr}^{k}+n^{k}$$

**Figure 10:**
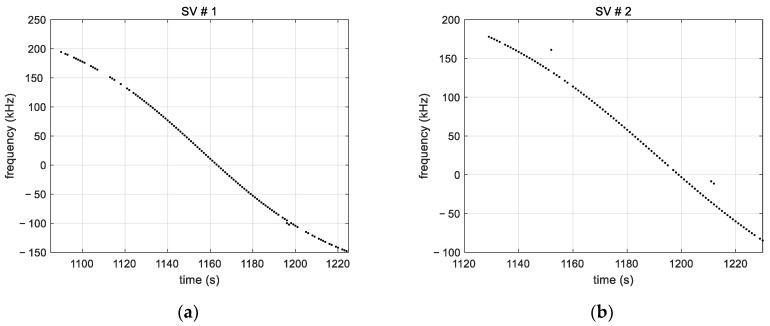
(**a**) Measured frequency for SV#1 after aggregating the multi-tone measurements of Figure 8a; (**b**) Measured frequency for SV#2 after aggregating the multi-tone measurements of Figure 8b.

$n^{k}$ is the noise of the aggregated frequency shift. Applied to the multi-tones frequencies given in Figure 8, this aggregation process leads to the results in Figure 10. As it can be seen, assumption (10) has been mostly met except for a few points. Since the tones’ frequency spacing, 44 kHz, is larger than the tracking loop bandwidth (100 Hz), the tones’ measurement noises are not correlated. Thus, for a given satellite, at times when there are measurements of more than one tone, the aggregation reduces the measurement noise by averaging. In addition, the tone aggregation fills in the measurement gaps of individual tones in degraded-like conditions of a Ku band signal received without a parabolic reflector.

Note that this model also applies if the tones are not centered exactly on *Fo*. Indeed, if the offset from *Fo* is the same for all satellites, it can be captured in the user clock drift in Doppler-based positioning as a common frequency error.

## 4. Multi-Epoch Positioning

**Figure 11:**
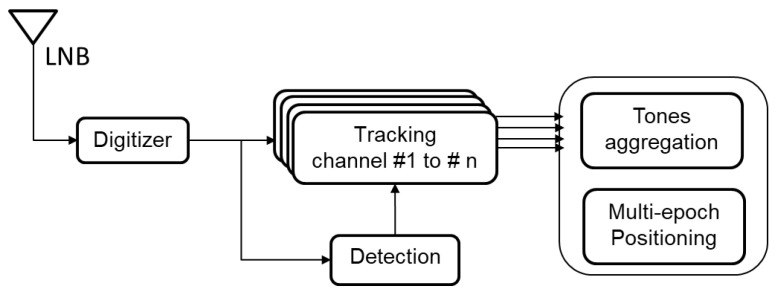
A block diagram showing the processing steps used to perform positioning from Starlink downlink tones.

As shown in Figure 11, Starlink downlink tones are down-converted by the LNB, then, the tones are acquired and tracked. The measured tone frequencies are then aggregated and used to estimate the user’s position. In this section, we first build the frequency observation model which will be simplified, then present the navigation algorithm and discuss the positioning results.

The range rate is the opposite sign of the Doppler shift times of the wavelength, i.e., ${\dot{\rho }}^{k}=-\lambda f^{k}$. We have shown in [21] that the *k*th-satellite range rate error at time t, $\Delta {\dot{\rho }}^{k}\left(t\right)={\tilde{\dot{\rho }}}^{k}\left(t\right)-{\hat{\dot{\rho }}}^{k}\left(t\right)$, which is the difference between the measured range rate and the predicted one, can be expressed as a function of the user’s position error $\delta r=r-\hat{r}$, the user’s velocity error $\delta v=v-\hat{v}$, the user clock bias error $\delta t=t-\hat{t}$, and the user clock drift error $\dot{\delta t}=\dot{t}-\hat{\;\dot{t}\;}$:

(16) $$\Delta {\dot{\rho }}^{k}\left(t\right)≅\left(\frac{{\left(v-v^{k}\right)}^{T}}{\left\|r-r^{k}\right\|}-u^{k}{}^{T}\left(v-v^{k}\right)\frac{u^{k}{}^{T}}{\left\|r-r^{k}\right\|}\;\right)\delta r+u^{k}{}^{T}\delta v+\frac{1}{c}\left(\frac{\left(u^{k}{}^{T}{\dot{r}}^{k}\right)u^{k}{}^{T}-{\dot{r}}^{k}{}^{T}}{\left\|r-r^{k}\right\|}\left(v-v^{k}\right)-u^{k}{}^{T}{\dot{v}}^{k}\right)c\delta t+c\dot{\delta t}-\lambda \delta f_{sv}^{k}+\eta_{\dot{\rho }}$$

where $\eta_{\dot{\rho }}$ is the range rate noise, and $u^{k}$ is the line-of-sight unit vector between the receiver and the satellite given by:

(17) $$u^{k}=\frac{r-r^{k}}{\left\|r-r^{k}\right\|}$$

In (16), we have added the term $\lambda \delta f_{sv}^{k}$, representing the *k*th-satellite clock drift range- rate, which we purposely not included in [21]. The tropospheric induced frequency error is assumed to have been compensated based on a model (for instance, the MOPS [32]). For clarity, we can set (16) in the form:

(18) $$\Delta {\tilde{{\dot{\rho }}^{k}}}_{i}=h_{r,i}^{k}\times \delta r+h_{v,i}^{k}\times \delta v+h_{c\delta t,i}^{k}\times c\delta t_{i}+c{\dot{\delta t}}_{i}-\lambda \delta f_{sv}^{k}{}_{i}+\eta_{\dot{\rho },i}$$

where the index i referes to time $t_{i}$, and the coefficients $h_{r,i}^{k}$, $h_{v,i}^{k}$, and $h_{c\delta t,i}^{k}$ can be easily deduced by term-to-term identification between (16) and (18).

### 4.1. A Simplified Frequency Error Model

The frequency model (18) has eight unknowns (3D position, 3D velocity, user clock bias, and user clock drift) plus as many unknowns as satellite clock drifts. The system is therefore undetermined. Moreover, the small number of satellites captured simultaneously is not very helpful. Therefore, we cannot perform point-solution batch positioning for a dynamic user. However, in the case of a static user, its position coordinates do not change over time and typically, a multi-epoch positioning is more practical in this situation. This positioning consists in using—for each tracked satellite—the measurements of several epochs simultaneously to make a state estimation. This is relevant because the fast motion of the LEO satellite makes successive measurements spaced a few seconds apart, sufficiently different for the observation of the unknowns.

We consider the multi-epoch positioning in which Doppler shifts of m epochs (numbered from 1 to m) of K tracked Satellites (k=1,..,K) are used to make an estimation of the unknowns. We assume that the user clock drift error is constant over a short time interval of m epochs, then ${\dot{c\delta t}}_{i}\approx c\dot{\delta t_{r}}$, for i=1,..,m. In addition, the receiver clock bias error at epoch i, $\delta t_{i}$ can be deduced from its value at the first epoch, $\delta t_{r}$, as

(19) $$c\delta t_{i}=c\delta t_{r}+\Delta t_{i,1}\times c{\dot{\delta t}}_{r}$$

where $\Delta t_{i1}=t_{i}-t_{1}$, i=1,..,m. Based on these assumptions, the range rate error of the *k*th-satellite at epoch i given by (18) becomes

(20) $$\Delta {\tilde{{\dot{\rho }}^{k}}}_{i}=h_{r,i}^{k}\times \delta r+h_{c\delta t,i}^{k}\times c\delta t_{r}+\left(1+h_{c\delta t,i}^{k}\Delta t_{i,1}\right)\times c{\dot{\delta t}}_{r}-\lambda \delta f_{sv}^{k}{}_{i}+\eta_{\dot{\rho },i}$$

For a static user, at each update based on m×K measurements (of m epochs and K satellites), the unknowns are: 3D user coordinates, r, user clock bias and user clock drift as $\delta t_{r}$ and ${\dot{\delta t}}_{r}$, respectively, both at the first epoch, and m×K satellite clock drift range rates $\lambda f_{sv}^{k}{}_{i},\;k=1..K,\;i=1..m$. There are a total of 5+m×K unknowns for m×K measurements. The system is therefore underdetermined regarding the number m of epochs that can be stacked. If we assume, in addition, that satellite clock errors are slightly varying over a short time interval and then considered constant i.e., $\delta f_{sv}^{k}{}_{i}=\delta f_{sv}^{k}$ for , i=1,..,m, the number of unknowns reduces to 5+K. By gathering measurements from several epochs, one should be able to estimate the user’s position. In this condition, the range-rate error model (20) becomes:

(21) $$\Delta {\tilde{{\dot{\rho }}^{k}}}_{i}=h_{r,i}^{k}\times \delta r+h_{c\delta t,i}^{k}\times c\delta t_{r}+\left(1+h_{c\delta t,i}^{k}\Delta t_{i,1}\right)\times c{\dot{\delta t}}_{r}-\lambda \delta f_{sv}^{k}+\eta_{\dot{\rho },i}$$

### 4.2. Positioning Algorithm

The expected accuracy of the satellites’ positions computed using TLE-SPG4 can be as high as a few km, and their velocities can be as high as a few m/s. As we showed in (6), the position and velocity errors of the satellites convert to frequency errors that are different between satellites. Thus, in the positioning filter based on the Doppler shift model presented above, these frequency errors will propagate into the satellite clock drift term, $f_{sv}^{k}$. We will investigate the possibility of absorbing the orbit-induced frequency error in the satellite clock drift state under the conditions of this study (i.e., a degraded signal level, inaccurate satellite orbits, unknown satellite clocks, and inaccurate measurement timestamps).

Consider an extended Kalman filter (EKF) with an error state vector δx consisting of the user’s position and velocity errors, the user clock bias and drift errors, and the frequency error of each tracked satellite:

(22) $$\delta x={\left[\delta r,c\delta t_{r},\;c\dot{\delta t_{r}},\lambda \delta f_{s}^{1},\ldots ,\lambda \delta f_{s}^{K}\right]}^{T}$$

In this state, each satellite brings a single additional unknown, instead of six unknowns if we had included the 3D position and velocity errors of each tracked satellite into the state. We can write the state dynamics as:

(23) $$\begin{array}{l} \dot{\delta }r=w_{v}\\ \dot{c\left(\delta t_{r}\right)}=c\left(\delta {\dot{t}}_{r}\right)+w_{c\left(\delta t_{r}\right)}\\ c\left({\ddot{\delta t}}_{r}\right)=w_{c\left(\delta {\dot{t}}_{r}\right)}\\ \dot{\lambda \left(\delta f^{k}\right)}=w_{\lambda \delta f} \end{array}$$

where $w_{v}$, $w_{c\left(\delta t_{r}\right)}$, $w_{c\left(\delta {\dot{t}}_{r}\right)}$, and $w_{\lambda \delta f}$ are centered white Gaussian processes of the user’s position error, the user clock bias error, the user clock drift error and the satellite-dependent frequency error, respectively. The system defined in (23) can be written as $\dot{\delta x}=F\delta x+W$, with F being the dynamic matrix and W being the system noise matrix with covariance Q=cov(W). The prediction covariance matrix is written $P=\phi P\phi^{T}+QT_{s}$, where $\phi =I+T_{s}F$ is the state transition matrix and $T_{s}$ is the time step of the prediction.

The state is updated by the measurement vector $\tilde{z}$ composed of range rates of K satellites (1,..,K) over m epochs (from i+1 to i+m). Thus, by stacking data of m epochs, we have:

(24) $$\tilde{z}={\left[{\tilde{{\dot{\rho }}^{1}}}_{i+1},\ldots ,{\tilde{{\dot{\rho }}^{K}}}_{i+1},\;\ldots ,{\tilde{{\dot{\rho }}^{1}}}_{i+m},\ldots ,{\tilde{{\dot{\rho }}^{K}}}_{i+m}\right]}^{T}$$

We assume that ${\tilde{{\dot{\rho }}^{k}}}_{i}$ has already been corrected for the tropospheric induced frequency error. We compute the measurement prediction according to:

(25) $$\hat{z}=\left[\begin{array}{c} -\lambda \left({\hat{f}}_{doppler}^{1}{}_{i+1}-{\hat{f}}_{s}^{1}\right)+c\hat{\dot{dt_{r}}}\\ .\\ .\\ -\lambda \left({\hat{f}}_{doppler}^{K}{}_{i+1}-{\hat{f}}_{s}^{K}\right)+c\hat{\dot{dt_{r}}}\\ .\\ .\\ -\lambda \left({\hat{f}}_{doppler}^{1}{}_{i+m}-{\hat{f}}_{s}^{1}\right)+c\hat{\dot{dt_{r}}}\\ .\\ .\\ -\lambda \left({\hat{f}}_{doppler}^{K}{}_{i+m}-{\hat{f}}_{s}^{K}\right)+c\hat{\dot{dt_{r}}} \end{array}\right]$$

where ${\hat{f}}_{doppler}^{k}{}_{j}$ for j=i+1 to i+m is computed based on the predicted user’s position $\hat{r}$ at time ${\hat{t}}_{j}=t_{j}+\hat{\delta t_{r}}$, and on the *k*th-satellite position and velocity at time ${\hat{t}}_{j}-\tau_{j}^{k}$, i.e., $r^{k}\left({\hat{t}}_{j}-\tau_{j}^{k}\right)$ and $v^{k}\left({\hat{t}}_{j}-\tau_{j}^{k}\right)$, where $\tau_{j}^{k}\approx \|\hat{r}\left(t_{j}\right)-r^{k}\left(t_{j}\right)\|\;/c$ is the propagation time of the signal between satellite and user’s positions.

By denoting innovation $\Delta z=\tilde{z}-\hat{z}$, the observation matrix H is defined such that Δz=Hδx+η, where η is the measure noise vector with covariance matrix R. The line of H corresponding to innovation $\Delta {\tilde{{\dot{\rho }}^{k}}}_{j}$ is $H\left(\Delta {\tilde{{\dot{\rho }}^{k}}}_{j}\right)=\left[h_{r,j}^{k},h_{c\delta t_{r},j}^{k},\left(1+{\hat{h}}_{c\delta t_{r},j}^{1}\Delta t_{j,1}\right),h_{\lambda \delta f}^{k}\right]$, with $h_{\lambda \delta f}^{k}$ being a 1×K vector whose *k*th-element is set to −1 and the remaining elements are set to 0. Every m epochs, the EKF filter gain is computed as $K=PH^{T}{\left(HPH^{T}+R\right)}^{-1}$ and the state and its covariance are respectively updated according to x=x+KΔz and P=P−KHP.

### 4.3. Results

We will investigate based on experimental data whether the frequency error state can absorb the frequency errors due to the satellite orbit error and the satellite clock drift. We collected 15 min of data in the 11,324–11,326 MHz band using the acquisition tool presented in Section 2.2, which we post-processed using the tracking tool presented in Section 2.3, to obtain tone frequency shifts that are provided to the multi-epoch positioning algorithm. The position was initialized 165 km away from the actual position of the recording location. The initial user clock drift and the initial frequency states are set to zero. The user time has an error of a few seconds.

**Figure 12:**
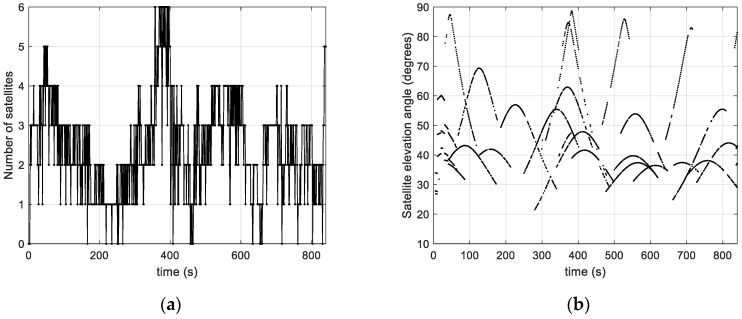
(**a**) Number of tracked satellites as function of time; (**b**) satellites’ elevation angles at measurement epochs.

Figure 12 gives the number of captured satellites as a function of time, and the elevation angles of the satellites from the observer’s point of view. We can see that the number of satellites captured reaches six, and that the satellites are mostly tracked above an elevation angle of 28° with a few satellites passing over the recording location (i.e., elevation angles near 90°). There are a few periods with no measurements at all. Using the acquisition tool of Section 2.2, the number of satellites actually tracked in this collection is therefore less than a fifth of the number of geometrically visible satellites.

**Figure 13:**
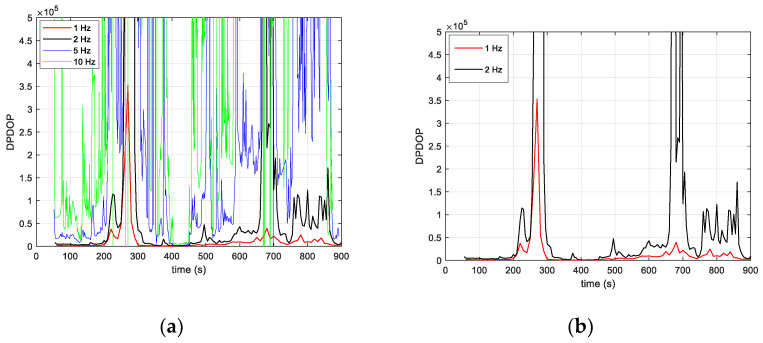
(**a**) Doppler position dilution of precision as a function of time for different values of the measurement rate (1 Hz, 2 Hz, 5 Hz and 10 Hz) in multi-epoch positioning; (**b**) zoom in for data rates 1 Hz and 2 Hz.

Figure 13 shows the Doppler position dilution of precision (DPDOP), a measure of satellite geometry. It is computed based on the observation matrix, H, and reflects the effect of the satellite geometry on the position state accuracy. The better the geometry of the satellite, the lower its value, and vice versa. A multi-epoch interval duration of m = 50 s is considered, and the DPDOP was calculated for different Doppler measurement rates between 1 Hz and 10 Hz. It can be seen that the DPDOP increases significantly with the measurement rate. This is because the satellites’ positions became too close, which led to unfavorable geometry. Strong peaks are observed for rates above 1 Hz, which is the consequence of a singularity in the observation model. The measurement rate at the input of the algorithm is therefore fixed at 1 Hz in order to have measurements referenced to satellite positions that are sufficiently spaced to avoid singularity problems in the algorithm. For a rate of 1 Hz, during periods when more than two satellites are available, the DPDOP is between 500 and 3500. Its value peaks during periods of low satellite visibility (near 250 s and 670 s, as can be seen in Figure 12a).

**Figure 14:**
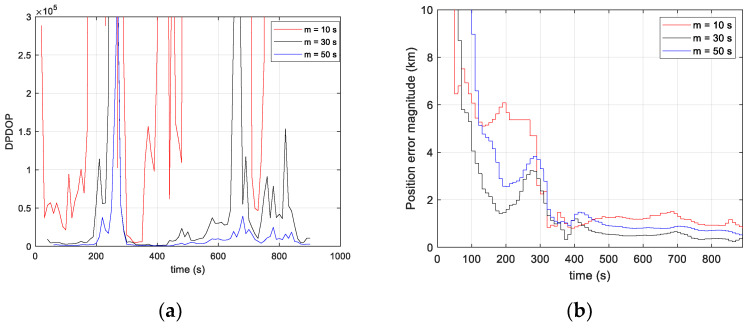
(**a**) Doppler position dilution of precision; (**b**) Position error magnitude. A comparison between three values of the multi-epoch interval duration, m.

The algorithm is run by trying different values of the multi-epoch interval (m = 10 s, 30 s and 50 s) in order to assess the effect of this parameter on the positioning accuracy. The positioning results as well as the DPDOP are shown in Figure 14.

The DPDOP increases as the number of stacked measurements decreases. In particular, for m = 10s, the algorithm often faces singularity problems due to the small number of satellites captured at the same time. After convergence, the position error stabilizes at ~1 km (3D) and is the least accurate for the tested values of m. This is due to the fact that the number of measurements accumulated over m = 10 s is not sufficient to better solve the unknowns. For m = 30 s, the DPDOP as well as the positioning result improves compared to m = 10 s. The position error after convergence is ~375 m (3D), and the convergence time is ~330 s. For m = 50 s, the position error, 705 m (3D), is worse than in the case of m = 30 s despite a better DPDOP. This is because in this case, the assumption of a constant frequency error state over the interval m is less tolerated by the measurement model, which degrades the positioning compared to m = 30 s. Thus, the duration of the multi-epoch interval, m, has to be chosen to reduce the DPDOP while satisfying the algorithm’s tolerance to the assumption of a constant frequency error state over the interval.

In the most favorable case of m = 30 s, the estimated user clock drift stabilizes at ~2.65 ppm, which is close to the specification of the clock frequency stability range (2 ppm). The algorithm cannot distinguish between the user clock drift and the satellite orbit and clock errors. For this reason, the user clock drift state converges to the average value of the sum of these errors over the measurements used. For comparison, we ran the filter by removing the frequency error from the filter state vector; we obtained a position error that exceeds 10 km (3D). This result is compliant with the expectation carried out in [21] showing that Doppler-based positioning is very sensitive to orbit errors. Therefore, the frequency error state has captured most of the satellites’ orbit and clock errors, preventing their total propagation into the user position state. The remaining position error is mainly due to measurement noise combined with weak satellite geometry.

## 5. Conclusions

We have investigated the feasibility of tracking Starlink downlink tones for opportunistic positioning in a practical situation, when signals are received without a parabolic reflector.

Based on the processing of real-life signals, the tones are detected and then tracked by means of an FLL-assisted-PLL. The results have shown that up to six satellites are tracked simultaneously with a C/No between 24 and 36 dB-Hz. In addition, without antenna reflector, tone tracking is often interrupted due to the weak strength of the tones combined with the oscillation in the tones’ magnitude. This suggests that in this situation, we cannot afford to track only a single tone for a satellite. Since such interruptions do not usually occur at the same time for tones of the same satellite, tones’ measurements have been aggregated in order to obtain the most continuous frequency shift measurement for each tracked satellite.

In order to determine the potential of Starlink downlink tones in positioning, the aggregated frequency shifts are then used to solve for the position of a static user. Multi-epoch positioning has been considered, in which frequency errors induced by satellite-imprecise orbits are captured in satellite-dependent frequency error states. The results showed that the measurement rate should not exceed 1 Hz to avoid any singularity in the observation model. In addition, the duration of the multi-epoch interval must satisfy a trade-off between good satellite geometry and meeting the assumption of constant satellite-dependent frequency error states.

The resulting positioning error, using 1 Hz rate measurements stacked over a 30 s duration, is 375 m. Note that this result is obtained with inaccurate satellite orbits (TLE file), inaccurate timing, and a relatively low signal strength. Optimizing the antenna and RF front end will improve both signal quality and satellite availability for better positioning with Ku-band signals.

## Figures and Tables

**Table 1:** Typical range limits of range dynamics and visibility for a static observer located at mid-latitude and using an elevation angle mask of 25°. The Doppler shift and the Doppler shift rates are given for 11,325 MHz, which is one of the Starlink signal frequencies. The used TLE file contains the orbits of nearly 3000 satellites.

	Unit	A Typical Range Limit
Range	km	400 to 1150
Doppler shift	kHz	−230 to 230
Doppler shift rate	kHz/s	<5
Rate of the Doppler shift rate	Hz/s^2^	<70
Number of satellites in view	-	16–35
Satellite visibility time	minutes	<4

**Table 2:** An example of the frequency error budget.

Frequency Error Source	Assumption	Frequency Error
Receiver clock drift	1 ppm	11.345 kHz
Satellite clock drift	0.01 ppm	0.11345 kHz
Troposphere	Elevation mask < 25°	<a few Hz
Time error	ΔT=1 s, $\dot{f^{k}}<5\;\mathrm{kHz}/s$	<5 kHz
Satellite and user position errors	$\|r-r^{k}\|=400\;\mathrm{km}$ $\|v-v^{k}\|=6.5\;\mathrm{km}/s$ $\|\Delta r-\Delta r^{k}\|=10\;\mathrm{km}$	<6.2 kHz
Satellite and user velocity errors	$\|\Delta v-\Delta v^{k}\|<5\;m/s$	<150 Hz
Noise	24–36 dB-Hz	<10 Hz

## Data Availability

Not applicable.
